# Evolution and Expression of the Expansin Genes in Emmer Wheat

**DOI:** 10.3390/ijms241814120

**Published:** 2023-09-15

**Authors:** Ming Li, Tao Liu, Rui Cao, Qibin Cao, Wei Tong, Weining Song

**Affiliations:** State Key Laboratory of Crop Stress Biology in Arid Areas, College of Agronomy, Northwest A&F University, Xianyang 712100, China; liming9256@outlook.com (M.L.); liutao22099@outlook.com (T.L.); 2021050127@nwafu.edu.cn (R.C.); 15610320650@163.com (Q.C.)

**Keywords:** emmer wheat, expansin gene family, evolution, expression pattern, haplotypes

## Abstract

Expansin proteins, a crucial class of intracellular proteins, are known to play a vital role in facilitating processes like cell wall relaxation and cell growth. Recent discoveries have revealed that expansin proteins also have significant functions in plant growth, development, and response to resistance. However, the expansin gene family, particularly in emmer wheat, has not been thoroughly studied, particularly in terms of evolution. In this study, we identified 63 *TdEXPs* and 49 *TtEXPs* from the latest genome versions of wild emmer wheat (WEW) and durum wheat (DW), respectively. The physicochemical properties of the encoded expansin proteins exhibited minimal differences, and the gene structures remained relatively conserved. Phylogenetic analysis categorized the proteins into three subfamilies, namely EXPA, EXPB, and EXLA, in addition to the EXLB subfamily. Furthermore, codon preference analysis revealed an increased usage frequency of the nucleotide “T” in expansin proteins throughout the evolution of WEW and DW. Collinearity analysis demonstrated higher orthology between the expansin proteins of WEW and DW, with a Ka/Ks ratio ranging from 0.4173 to 0.9494, indicating purifying selection during the evolution from WEW to DW. Haplotype analysis of the expansin gene family identified five genes in which certain haplotypes gradually became dominant over the course of evolution, enabling adaptation for survival and improvement. Expression pattern analysis indicated tissue-specific expression of expansin genes in emmer wheat, and some of these genes were quantified through qRT-PCR to assess their response to salt stress. These comprehensive findings present the first systematic analysis of the expansin protein gene family during the evolution from WEW to DW, providing a foundation for further understanding the functions and biological roles of expansin protein genes in emmer wheat.

## 1. Introduction

As an essential structure of the cell, the cytoderm not only glues cells together and sustains shape, but also enhances the essential mechanical strength of plant cells [1,2]. It also provides the primary critical screen for plant resistance and defense, which in turn assures the development of plant tissues and organs [3,4,5,6,7]. In 1992, McQueen-Mason S et al. first revealed that expansin proteins play a major function in the process of cytoderm stretch [8]. The protein in the hypocotyl cell wall of cucumber was identified, which allows heat-deactivated cell walls to reinstate stretching activity [9,10]. Thus, the mechanism and influences of expansin proteins in plants have been studied intensively.

Expansin genes have been discovered to be significant in plant growing development and resistance to stresses [5,6,11,12,13]. Cloned *BrEXLB1* gene in *Brassica rape* was overexpressed in *Arabidopsis*, which increased the size of the root elongation zone and induced primary root growth for *Arabidopsis* transgenic plants, yet it caused a decrease in the germination rate of seeds. However, the overexpressed transgenic strains for *Brassica rape* were shown to be positively associated with drought tolerance and photosynthesis during the vegetative growth period [14]. *IbEXP1* from *Ipomoea batatas* was heterologously overexpressed in *Arabidopsis thaliana* and found that plants of the overexpressed strains grew faster, had an increased number of rosette leaves, and had larger and more numerous seeds than wild-type, which caused an increasing yield per plant [15]. The *PpEXP1* gene from *Poa pratensis* was overexpressed in tobacco and it was found that under heat stress conditions at 42 °C, the transgenic tobacco strains displayed low cell structure damage, increased chlorophyll content, cytoplasmic rate, antioxidant enzyme activity, and seed germination rate. It showed potential positive roles in improving plant heat tolerance [16].

Expansins are a multi-gene family that is widely distributed in land plants [17,18]. Expansin family genes have been found to contain two domains (DPBB_1 and Pollen_allerg_1), and show four subfamilies: EXPA (α-expansin), EXPB (β-expansin), EXLA (expansin-like A), and EXLB (expansin-like B) [19,20,21]. The differential number of expansin genes contained in many plants, such as 34 in *Arabidopsis thaliana*, 38 in rice (*Oryza sativa*), 37 in sweet potato (*Ipomoea batatas*), 82 in moso Bamboo (*Phyllostachys edulis*), 56, 58, and 60 in *Brassica rapa*, *Brassica oleracea* and *Brassica nigra*, 30 in jujube (*Ziziphus jujuba*), 36 in potato (*Solanum tuberosum*), 75 in wild soybean (*Glycine soja*), and so on [22,23,24,25,26,27,28]. In bread wheat (*Triticum aestivum*), the genes number of expansin family has been found for several articles to be 87, 128, 241, and 104 [29,30,31,32]. In *Triticum turgidum*, chen et al. also found 50 expansin genes [32]. However, the expansin family proteins in wild emmer wheat is very unclear. Durum wheat and wild emmer wheat belong to tetraploid species with the chromosome (2n = 4x = 28) and the chromosome haplotype AABB [33,34]. The domestication of wild emmer wheat has produced modern durum wheat, whereas the latter is now cultivated in some regions of the world [35]. In this study, whole-genome identification of the expansin family proteins was performed with newly available reference genomic information, and then features such as phylogenetic relationships, gene structure, collinearity, selection pressure, and gene codon bias were explored. Additionally, gene expression patterns were also studied. The inquiry of these data reveals the selection and conservation of expansin proteins during the domestication of wild emmer wheat to durum wheat. It is the inaugural expansin gene family in wild emmer wheat and durum wheat and would provide an outstanding rationale for further gene functional studies.

## 2. Results

### 2.1. Identification of Expansin Gene Family in Wild Emmer Wheat and Durum Wheat

**Figure 1:**
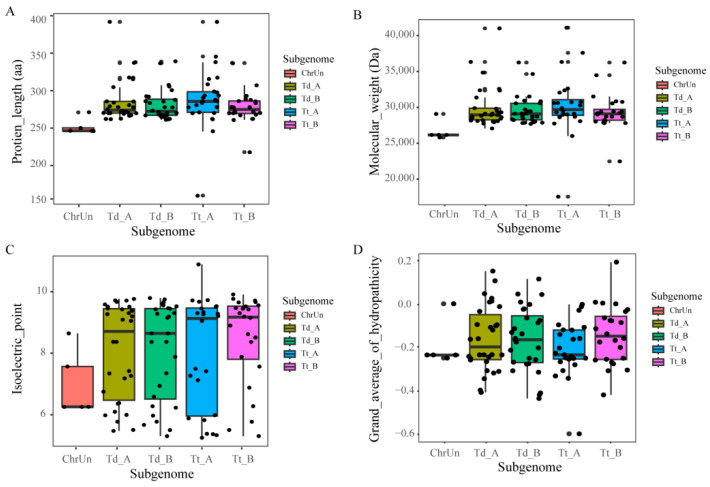
Statistics and analysis of expansin gene sequences on subgenomes. (**A**) The protein sequence length of expansin genes family in *Triticum dicoccoides* and *Triticum turgidum*; (**B**) the molecular weight of expansin genes family; (**C**) the isoelectric point of expansin genes family; (**D**) the grand average of hydropathicity in expansin genes family, the Td-A and Td-B shows subgenome A and B in wild emmer wheat, Tt-A and Tt-B shows subgenome A and B in durum wheat, ChrUn show unplaced scaffolds.

Blastp and HHM modeling methods were used to assay the Expansin gene family (both of them contained two domains (DPBB_1 and Pollen_allerg_1)) in wild emmer wheat and durum wheat. In wild emmer wheat (Figure 1 and Table S1), we identified 63 genes (sequentially named *TdEXP1-63*), of which 30 were on subgenome A (Td-A), 28 on subgenome B (Td-B), and 5 were localized on unplaced scaffolds. The sequences of proteins were between 249 and 397 aa, with an average amino acid length of 284 aa, the isoelectric point (pI) between 4.77 and 9.53, average 7.62, molecular weight (Mw) between 26,463.01 and 42,484.70, average 30,407.00, and grand average of hydropathicity (GRAVY) between 0.441 and 0.125, average 0.179.

In durum wheat (Figure 1 and Table S2), we characterized 49 genes (named *TtEXP1-49*), 25 on subgenome A (Tt-A), and 24 on subgenome B (Tt-B). The protein sequences were between 161 and 397 aa, with an amino acid average of 287 aa in length, the pI between 4.71 and 10.69, average 7.88, the MW (Da) between 17,762.89 and 42,581.86, average 30,689.78, the GRAVY in between −0.598 and −0.165, and average −0.192.

Overall, the number of expansin genes was found to be from 63 to 49 in the wild emmer wheat to durum wheat. In the A subgenome, Td-A to Tt-A shows 30 to 25. In the B subgenome, Td-B to Tt-B shows 28 to 24. As it is known, the protein of expansin family is still relatively evolutionary conservative.

### 2.2. Phylogenetic Relationship Analysis of Expansins

**Figure 2:**
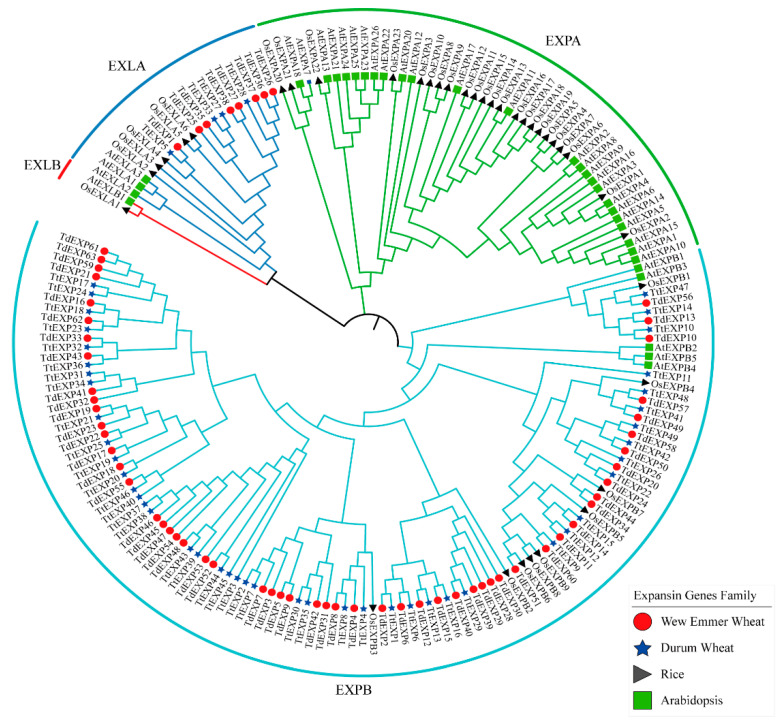
Phylogenetic relationship analysis of the expansin genes by MEGA 9.0 software.

To know the phylogenetic relationship of expansin in this study, the evolutionary tree that also joined expansins of *Arabidopsis* and rice was constructed, including 63 and 49 wild emmer wheat (*TdEXPs*) and durum wheat (*TtEXPs*), respectively, 34 *Arabidopsis* (*AtEXPs*), and 38 rice (*OsEXPs*) [22]. In Figure 2, the expansin proteins were classified into four subfamilies, EXPA, EXPB, EXLA, and EXLB. Among them, EXPB subfamily member 114, including 55 *TdEXPs*, 45 *TtEXPs*, 5 *AtEXPBs,* and 9 *OsEXPBs*, followed by EXPA subfamily member 48, including 24 *AtEXPAs* and 24 *OsEXPAs*, among EXLA subfamily members 20, 8 *TdEXPs*, 4 *TtEXPs*, 3 *AtEXLAs,* and 5 *OsEXLAs*; there were not any genes for *TdEXP* and *TtEXP* found in the EXLB subfamily members.

### 2.3. Codon Usage Bias Analyses

**Figure 3:**
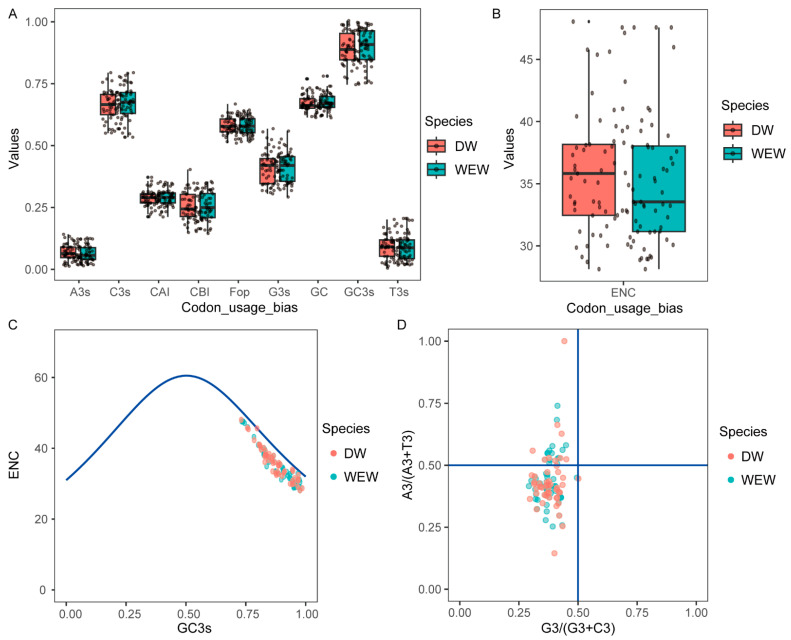
The codon usage bias analyses of expansin gene family in wild emmer wheat (WEW) and durum wheat (DW). (**A**) Characterization of codon usage bias; (**B**) ENC analysis; (**C**) ENC-plot curves of the codons, the blue solid curve shows the expected positions of the genes which are determined only by GCs; (**D**) PR2-plots analysis, blue lines were x = 0.5 and y = 0.5.

The average frequency of A3s, C3s, G3s, T3s, and GC3s in wild emmer wheat and durum wheat (Figure 3A and Table S3), respectively, were 5.69% and 6.24%, 65.76% and 64.72%, 40.17% and 40.23%, 8.11% and 8.61%, and 88.33% and 87.47%. Meanwhile, the total average GC content of the expansin family is 66.08% and 65.67%. These results show that the GC3 and GC of *TdEXPs* and *TtEXPs* genes in wild emmer wheat and durum wheat have higher content, such as *Arabidopsis* [36]. ENC values mean effective codon number (Figure 3B), ENC ranged from 28.13 to 47.57 and 28.13 to 48.04, and the averages were about 35.24 and 35.89. CAI values indicate the codon adaptation index, the average of which was about 0.281 and 0.278. The ENC-plot shows the scatter plot that takes GC3s value as the X and ENC value as the Y (Figure 3C). The majority of the gene members for both *TdEXPs* and *TtEXPs* were distributed to the lower right of the predicted curve, but these points in no way deviated more from the curve, suggesting that the codon preferences of *TdEXPs* and *TtEXPs* may be affected by base mutations and other factors. The relationship between A/G and T/C in the third codon of the amino acids that form *TdEXPs* and *TtEXPs* was analyzed by PR2 (Figure 3D). Both *TdEXPs* and *TtEXPs* were distributed to the left of the vertical coordinate (center line on 0.5) and most of them were located in the lower left; these show that in the expansin family C has a higher frequency of usages than G.

### 2.4. Gene Structure and Protein Motifs Analysis of Expansins

**Figure 4:**
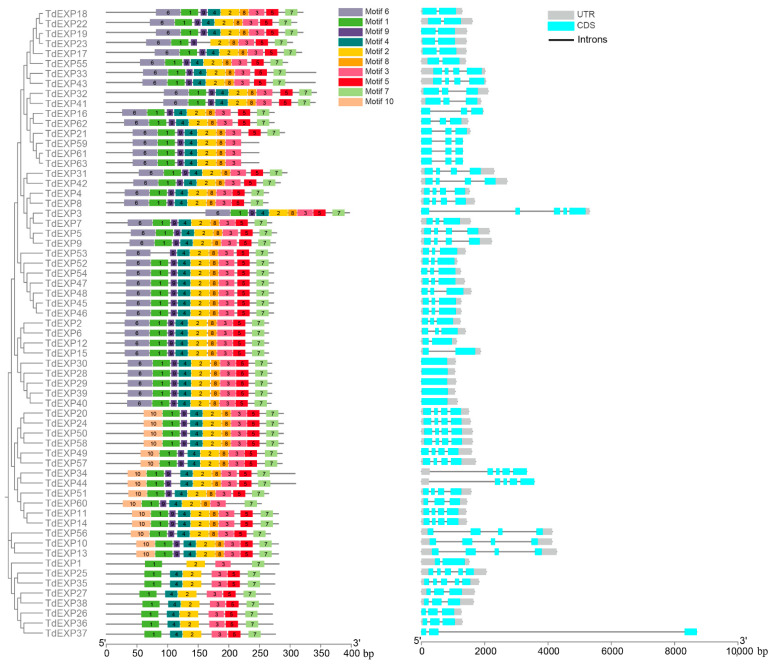
Gene motifs and structure analysis of expansin families in wild emmer wheat.

To better understand the similarities, differences, and distribution of gene structure and protein motifs, the gene structure and conserved motifs were mapped with TBtools. In wild emmer wheat (Figure 4), the numbers of exon–intron were found to be between 1 and 5 in exons for the expansin family with about 7.94% of genes that included 1 exon, 14.29% of those with 2 exons, 38.10% of those with 3 exons, 34.92% of those with 4 exons, and 4.76% of those with 5 exons. Further analysis of the MEME motif constructs (1 to 10) for expansin protein sequences showed that motif 10 was identified in only 15 encoded proteins, motif 6 was identified in only 23 coding proteins, motif 4 was found in 61, and motif 1 was unidentified in 62. However, motif 2, which exhibits the more abundant and highly conserved motifs, are recognized in all expansin families.

**Figure 5:**
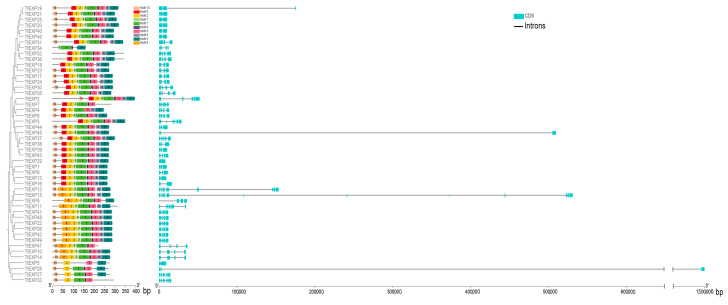
Gene motifs and structure analysis of expansin families in durum wheat.

In durum wheat (Figure 5), the number of exons was discovered to be from 1 to 11 (except 7 and 9). There was one exon (about 2.04%) in *TtEXP12*, *TtEXP15*, *TtEXP29*, genes with two exons for about 14.29%, three exons for about 28.57%, four exons for about 38.78%, five and six exons for about 6.12%, and *TtEXP12* and *TtEXP15* had eight and eleven exons (about 2.04%). Analysis of the MEME motifs (1 to 10) in the protein sequences showed that motif 9 was detected in only 13 coding proteins (26.53%), motif 5 was found in only 30 coding proteins (61.22%), motif 6 was found in only 40 coding proteins (81.63%), motif 7 was found in only 44 coding proteins (89.80%), motifs 1, 2, and 3 were found in 48 coding proteins (95.92%), and motif 4 and motif 9 were found in 47 coding proteins (97.96%). Most of the relatively conserved motif units may be present in the expansin family.

### 2.5. Syntenic Relationship Analyses of Expansin Gene Family

**Figure 6:**
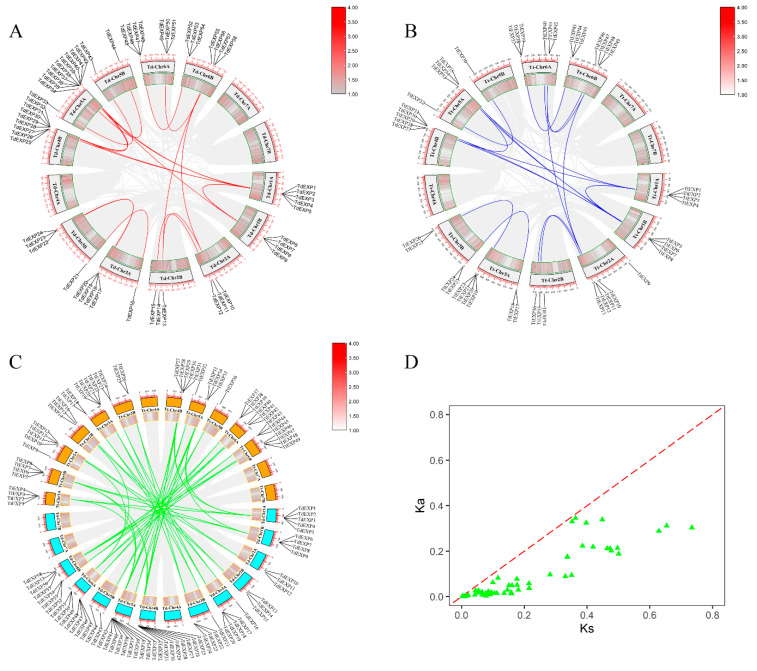
The syntenic relationship analyses of expansin gene family in wild emmer wheat and durum wheat. (**A**) Homologous genes in wild emmer wheat; (**B**) homologous genes in durum wheat; (**C**) homologous genes between wild emmer wheat and durum wheat; (**D**) Ka/Ks analysis between wild emmer wheat and durum wheat; green triangles show Ka/Ks values, red dotted lines show Ka/Ks = 1.

Analysis of co-linearity within (inter)species genomes could reflect genome-wide duplication events as species undergo evolutionary historical processes. In Figure 6A–C, homologous genes in wild emmer wheat (Td-Td) and durum wheat (Tt-Tt), and homologous genes between wild emmer wheat and durum wheat (Td-Tt) were performed using the MCscan program in TBools for expansin family (Table S4). We identified 27 pairs of homologous of 63 expansin family genes in the Td-Td (Figure 6A). Although mean, 20 pairs of homologous were determined for 49 family genes in the Tt-Tt (Figure 6B). Even more significantly, 71 pairs of homologous genes were obtained between wild emmer wheat and durum wheat (Td-Tt) (Figure 6C).

The Ka/Ks ratio analysis may relay the role of selection pressure on protein-coding genes. The relationship between Ka values and formula T = (Ks/2λ) × 10^−6^ Mya was also taken to assess the divergence time of replication events. We calculated Ka and Ks values for duplicated genes in the gene family in wild emmer wheat and durum wheat (Table S5); these results showed that Ka ranged from 0.0015–0.3449, Ks in 0.0034–0.7340, and Ka/Ks in 0.4173–0.9494, with an average of 0.3493 (Table S5). Ka/Ks values were less than 1 (Figure 6D), which refers to the expansin family being exposed to purifying selection. The divergence time in the expansin family gene of wild emmer wheat to durum wheat was approximately 0.26–56.46 Mya.

### 2.6. Haplotype Analysis of Expansin Gene Family in Wild Emmer Wheat and Durum Wheat

**Figure 7:**
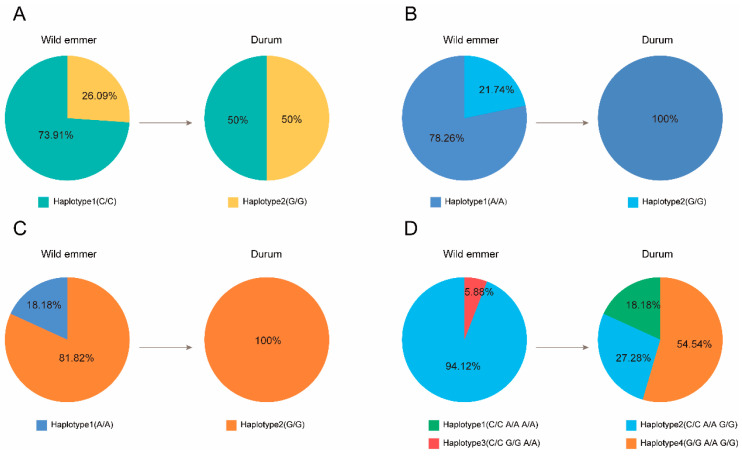
Haplotype frequencies of five genes in expansin family in wild emmer wheat and durum wheat. (**A**) The frequency of each haplotype in wild emmer wheat and durum wheat for *TtEXP1* and *TdEXP2*; (**B**) the frequency of each haplotype in wild emmer wheat and durum wheat for *TtEXP3*; (**C**) the frequency of each haplotype in wild emmer wheat and durum wheat for *TdEXP5*; (**D**) the frequency of each haplotype in wild emmer wheat and durum wheat for *TdEXP1*.

The haplotypes of the expansin gene family in wild emmer wheat and durum wheat were obtained using public resequencing data. Five genes from wild emmer wheat and durum wheat, co-localized on chromosome 1A, were successfully characterized. Among them, three genes (*TdEXP1*, *TdEXP2*, and *TdEXP5*) were distributed in wild emmer wheat, whereas two genes (*TtEXP1*, *TtEXP3*) were found in durum wheat (Table S6). Moreover, *TtEXP1* and *TdEXP2* share the same SNP loci (Table S6). Then, we calculated the frequency of each haplotype across five genes from 28 accessions in wild emmer wheat to 13 accessions in durum wheat revealing the dynamic change during the domestication process. Our findings demonstrated that there were two haplotypes for both *TtEXP1* and *TdEXP2*, named G/G and C/C. Within wild emmer wheat, haplotype 1 (C/C) makes up 73.91% and haplotype 2 (G/G) accounts for 26.09%. In contrast, the frequency of haplotype 1 (C/C) decreased to 50%, as haplotype 2 (G/G) increased to 50% in durum wheat (Figure 7A). In *TtEXP3*, haplotype 2 (A/A) was lost within durum wheat, and all of the durum wheat accessions were haplotype 1 (G/G) (Figure 7B), suggesting that haplotype 1 (G/G) underwent strong selection during domestication. Similarly, *TdEXP5* experienced the loss of haplotype 1 (A/A) and was a strong selection for haplotype 2 (G/G) within durum wheat (Figure 7C), further indicating that *TtEXP3* and *TdEXP5* possess genetic diversity within wild emmer wheat that has been lost throughout domestication. However, *TdEXP1* contains five haplotypes composed of three single nucleotide polymorphism (SNP) sites. Although wild emmer wheat contains only two haplotypes, durum wheat possesses three haplotypes. Haplotype 3 (C/C G/G A/A) was lost during domestication (Figure 7D). Although haplotype 2 (C/C A/A G/G) was preserved, its frequency decreased from 94.12% to 27.28%. Additionally, haplotypes 1 (C/C A/A A/A) and haplotypes 4 (G/G A/A G/G) were added, with haplotype 4 (G/G A/A G/G) having a higher frequency than haplotypes 1 (C/C A/A A/A) and haplotypes 2 (C/C A/A G/G). Through genetic variation analysis, it was found that a C nucleotide at position 364656606 bp on chromosome 1A of haplotype 4 (G/G A/A G/G) mutated to a G nucleotide, changing the codon from TAC to a stop codon TAG. This resulted in early termination of the amino acid translation process and the loss of the DCBB1 motif.

### 2.7. Expression Analyses of Expansin Gene Family

**Figure 8:**
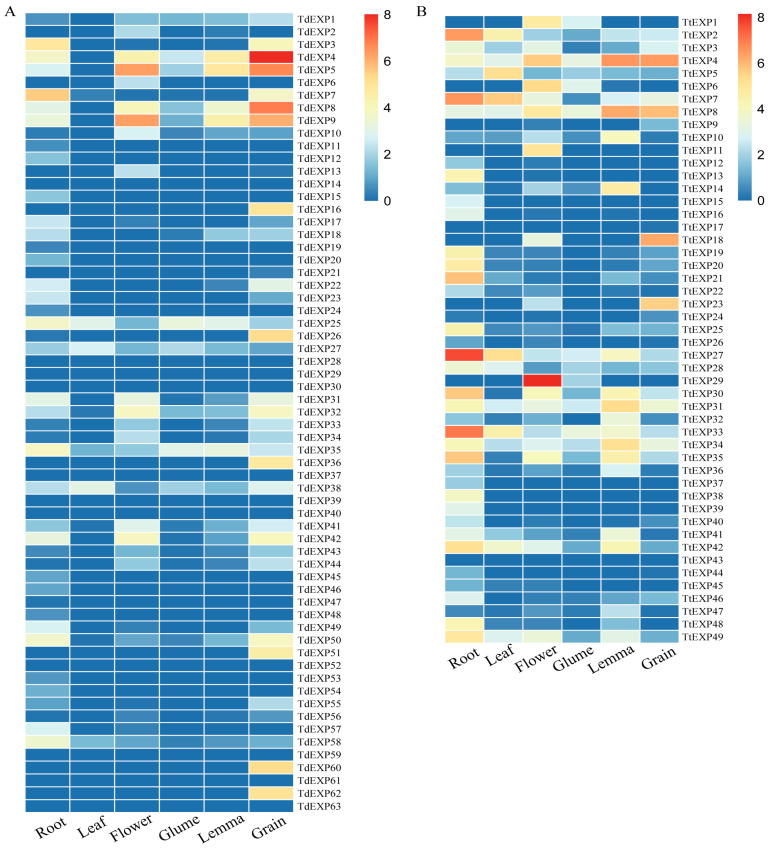
The expression patterns analysis of different tissues. (**A**) Expression of expansin family genes in wild emmer wheat; (**B**) expression of expansin family genes in durum wheat.

For constructing the expression of the expansin genes, we analyzed the expression levels of genes in different tissues (Table S7). As shown in Figure 8A, the majority of genes in WEW were expressed in different tissues, with relatively more numbers of genes expressed in root and grain. *TdEXP4, TdEXP5,* and *TdEXP8* were comparatively more highly expressed in grain. *TdEXP3* and *TdEXP7* were relatively more expressed in the root. However, *TdEXP14*, *TdEXP28*, *TdEXP29*, *TdEXP30*, *TdEXP37*, *TdEXP39*, *TdEXP40*, *TdEXP52*, *TdEXP59*, *TdEXP61,* and *TdEXP63* were unexpressed in any of the tissues. Although there were some genes that showed specific expression, *TdEXP11*, *TdEXP12*, *TdEXP15*, *TdEXP19*, *TdEXP20*, *TdEXP24*, *TdEXP45*, *TdEXP46*, *TdEXP47*, *TdEXP48*, *TdEXP53,* and *TdEXP54* were only expressed in the root; this indicates that *TdEXPs* may have an essential role in the roots of WEW.

It was also found in DW (Figure 8B) that most genes were expressed in different tissues, of which the quantity of genes has been expressed in root. *TtEXP2*, *TtEXP7*, *TtEXP27*, and *TtEXP33* in root were expressed relatively higher. *TtEXP29* were expressed relatively higher in flower. Meanwhile, *TtEXP4* and *TtEXP18* had relatively higher expression in the grain. However, *TtEXP43* was not expressed in all tissues. *TtEXP11* was only expressed in flower and *TtEXP17* was only expressed in grain.

**Figure 9:**
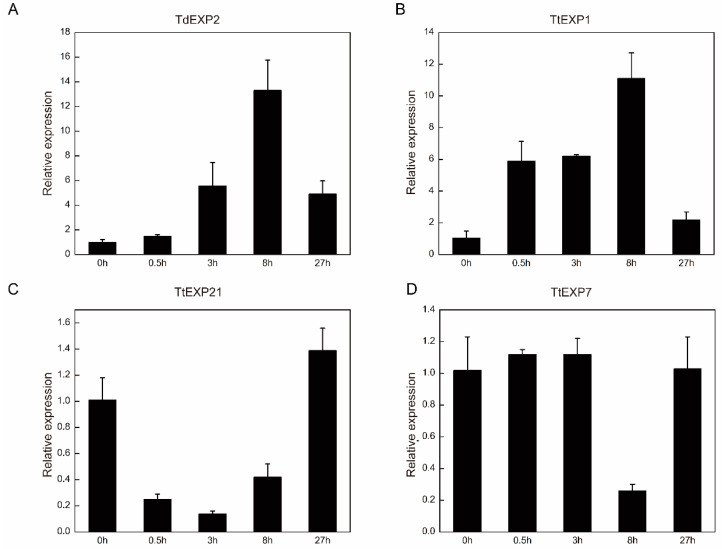
The qRT-PCR analysis of four expansin family genes in salt treatment. (**A**) Relative expression of *TdEXP2* gene under different time nodes in wild emmer wheat; (**B**–**D**) relative expression of three genes under different time nodes in durum wheat, *TtEXP1*, *TtEXP21*, and *TtEXP7*, separately.

To identify the expansin family members involved in the salt response, we further selected one gene in wild emmer and three genes in durum to validate their expression by qRT-PCR analysis in different time nodes in salt treatment. Relative expression results of *TdEXP2* and *TtEXP1* were shown to upregulate the expression trend from 0 h to 8 h, yet the expression level sharply declined at 27 h (Figure 9A,B). Interestingly, the tendency of expression to decline and then rise was different from the other three in the *TtEXP21* gene (Figure 9C). Ultimately, the relative expression results of *TtEXP7* significantly decrease at 8 h (Figure 9D). The experiment results show that their expression responds to salt resistance.

## 3. Discussion

The expansin protein gene family has been previously discovered in several other species to be essential for steering plant growth and development [11,22,25,37,38,39]. Wild emmer wheat and durum wheat provide an opportunity to offer an essentially significant contribution to the germplasm resources of wheat [40,41,42]. It is necessary to accelerate the research on the relevant genetic information and characterization in the expansin gene family. In this study, 49 members of the expansin gene family were identified in durum wheat. This result was close to the number of expansin genes, found in a previous study [32]. Meanwhile, 63 expansin family genes were first recognized in wild emmer wheat. This will facilitate that expansin gene features were deeply understood in wheat.

Gene duplication and loss play an increasingly critical role in evolution and amplification, and there is a high-level homology of the genes on the genomes of wild emmer wheat and durum wheat [34,43,44]. In this study, there were 27 pairs of homologous in 63 expansin genes of wild emmer wheat; some genes shown duplication events, for example, that homologous genes of *TdEXP1* had *TdEXP25* and *TdEXP35*, and *TdEXP2* had *TdEXP6* and *TdEXP29*. Similar results were found in durum wheat; for example, the homologous genes of *TtEXP1* had *TtEXP6* and *TtEXP29*, and *TtEXP5* had *TtEXP27* and *TtEXP33*. Between wild emmer wheat and durum wheat, *TdEXP40 in* WEW were homologous to three genes (*TtEXP1*, *TtEXP6*, *TtEXP29*) in DW, for example, *TdEXP1* and *TtEXP5*, *TtEXP27*, *TtEXP19*, *TdEXP42,* and *TtEXP4*, *TtEXP8*, and *TtEXP35*. Meanwhile, homologous genes of *TdEXP28* were *TdEXP39* in WEW, and *TdEXP28* was homologous for *TtEXP6* in DW; however, *TdEXP39* was not found in durum wheat. These findings show the existence of gene duplication and loss of expansin genes during the evolution process of wild emmer wheat and durum wheat. Chen et al. also found that expansin genes in wheat displayed gene duplication and loss [32]. More significantly, it was found that the Ka values of the orthologs in the expansin protein family between wild emmer wheat and durum wheat were in the range of 0.0034–0.7340, 0.0015–0.3449 for Ks, and 0.4173–0.9494 for Ka/Ks. With Ka/Ks values less than 1, the expansin protein family was selected for purification. The divergence between *TdEXPs* and *TtEXPs* occurred in about 0.26–56.46 Mya. The replication evolution time of paralogous pairs in *PeEXs* in moso bamboo was about 7–12 million years ago (Mya), and the replication evolution time of orthologous pairs between *PeEXs* and *OsEXs* in moso bamboo and rice was about 0.40–0.50 million years ago (Mya) [24].

Codon usage bias analysis is a fitting strategy to identify the primary evolutionary forces in different species [45,46,47,48]. In this study, it is that the average frequency of GC3 in wild emmer wheat and durum wheat, respectively, were 88.33% and 87.47%. These results indicate that the GC contents in the expansin family of WEW and DW may be a factor influencing codon usage. Zhang et al. found that expansin genes showed higher GC values in monocot plants [49]. The research suggested that there is a stronger bias in codon use when the mean ENC is less than 35 [50,51]. In wild and durum wheat, the ENC was 35.24 and 35.89, respectively. They were close to 35, indicating that *TdEXPs* and *TtEXPs* show some codon preference. PR2 analysis revealed that *TdEXPs* and *TtEXPs* were greatly influenced by the C frequency in the 3rd codon of amino acids. The codon adaptation index (CAI, 0–1.0) was used to estimate the adaptation of genes to codon usage of highly expressed genes [52,53]. Higher CAI values represent a potentially higher level of expression and potentially greater codon usage bias [54,55]. However, the CAI in the WEW has a range of 0.204–0.344 with an average value of 0.2808. The CAI of DW has a range of 0.205–0.362 with an average value of 0.2785. CAI values are relatively weak, which implicitly means that the expression level of the expansin gene family was relatively low. Meanwhile, in WEW, the larger CAI values of *TdEXP4* (0.344) and *TdEXP8* (0.338) displayed higher expression levels in grain. In DW, *TtEXP4* (0.347) and *TdEXP29* (0.333) with larger CAI values showed higher expression levels in the lemma and flower, respectively. During the domestication of tetraploid wheat, some haplotypes were lost, suggesting that it is the dominant haplotype in durum wheat. For instance, *TtEXP3* and *TdEXP5* genes only have one haplotype in durum wheat, whereas there are two haplotypes in wild wheat. There are novel haplotypes that account for more than half of the total (54.54%) and less ratio (18.18%) in *TdEXP1,* respectively. Therefore, we speculate that haplotype 4 (G/G A/A G/G) of *TdEXP1* may be a dominant haplotype in tetraploid wheat breeding. However, different tissue expression patterns were found that a higher number of genes were expressed in root and grain in WEW. Genes with a higher number of expressions were shown in root in DW. This also corroborates previous studies which also found that the expansin family plays a role in root and reproductive growth. Certainly, many more data are needed for further and deeper exploration in the future.

## 4. Materials and Methods

### 4.1. Wheat Identification of Expansin Genes Family in Wild Emmer Wheat and Durum Wheat

To identify the expansin gene, the sequences and annotation information of wild emmer wheat and durum wheat were downloaded from https://ftp.ncbi.nlm.nih.gov/genomes/all/GCF/002/162/155/GCF_002162155.2_WEW_v2.1/, accessed on 2 October 2022 (Genome assembly WEW_v2.1, *Triticum dicoccoides* (wild emmer wheat)) [35], and https://ftp.ncbi.nlm.nih.gov/genomes/all/GCA/900/231/445/GCA_900231445.1_Svevo.v1/, accessed on 2 October 2022 (Genome assembly Svevo.v1, *Triticum turgidum* subsp. *durum* (durum wheat)) [56]. We performed the identification of expansin family members by two methods. Firstly, scanning the whole genome protein sequence of wild emmer wheat and durum wheat were analyzed by query sequences (1 × 10^−5^ cut-off E-value) for BLASTP with expansin protein in *Arabidopsis thaliana* (https://www.arabidopsis.org/; accessed on 4 October 2022). Secondly, sequences of PF01357 (Pollen_allerg_1) and PF03330 (DPBB_1) of expansin family protein were downloaded from https://pfam.xfam.org/ (accessed on 4 October 2022). Then, we searched protein sequences in wild emmer wheat and durum wheat based on the hidden Markov model (HMM) by HMMSEARCH from hmmer 3.3.2 software (threshold of e-values < 10^−5^) [57]. Finally, the candidate sequences were presented to NCBI-CDD (https://www.ncbi.nlm.nih.gov/Structure/bwrpsb/bwrpsb.cgi; accessed on 4 October 2022) and PFAM (https://www.ebi.ac.uk/Tools/pfa/pfamscan/; accessed on 4 October 2022) to identify the expansin family domain. Only protein sequences containing both DPBB_1 and Pollen_allerg_1 structural domains were shown as expansin family genes. For subsequent analysis and study, only one protein sequence for each gene was retained. The physical–chemical parameters (PI, Mw, and GRAVY) of the characterized expansin family protein were obtained by the Expasy database (https://web.expasy.org/protparam/; accessed on 4 October 2022).

### 4.2. Phylogenetic Analysis of Expansin Family Genes

Collected, the expansin family sequences in *Arabidopsis* and rice were used together for evolutionary tree analysis using MEGA 9.0 software [22]. The amino acid sequences were aligned by ClustalW in MEGA 9.0, and the phylogenetic tree was constructed using the Neighbor-Joining method with the Bootstrap value of 1000, with other parameters set by default. The obtained evolution tree file was beautified and displayed on the online tool (iTOL, https://itol.embl.de/; accessed on 15 October 2022).

### 4.3. Codon Bias Analysis

Gene codon usage bias is closely associated with gene expression. We analyzed the codon bias of expansin family sequences using codonW (1.4.2) software (https://codonw.sourceforge.net/; accessed on 15 October 2022) with default parameters, mainly including CUB (codon usage bias), ENC (effective number of codon), CBI (codon bias index), CAI (codon adaptation index), and Fop (frequency of optional codon). PR2 (parity rule 2) showed x = G3/(G3 + C3) and y = A3/(A3 + T3), ENC (exp) = 2 + GC3s + 29/[GC3s ^2^ + (1 − GC3s)^2^].

### 4.4. Gene Structure and Conserved Motifs Analysis

For conserved motifs analysis, the protein sequences were submitted on motif-based sequence analysis tools (MEME, https://meme-suite.org/meme/tools/meme; accessed on 9 December 2022) to analyze motifs of expansin; the number of motifs is fixed to 10. The exon–intron structures of the genes were established based on the genome GFF information. These results are actualized by the TBtools tool (v1.106).

### 4.5. Synteny and Gene Duplication Analysis

To further understand gene synteny and duplication of expansin family genes in wild emmer wheat and durum wheat. This analysis was performed using the Step MCScanX program from the TBtools (v1.106) and the visualization of the results was performed via TBtools (v1.106) [58]. Ka/Ks indicate the ratios between the non-synonymous substitution rate (Ka) and the synonymous substitution rate (Ks) of two protein-encoding genes [59]. Ka and Ks values between homologous genes were obtained through Ka/Ks calculator within the TBtools. Ks has a connection with the time to replication events; the formula T = (Ks / 2λ) × 10^−6^ Mya (λ = 6.5 × 10^−9^) was utilized to acquire the divergence time (T) [60].

### 4.6. Haplotype Analysis of Expansin Gene Family

The genotype data of tetraploid emmer wheat were obtained from Genome Variation Map (https://bigd.big.ac.cn/gvm; accessed on 9 April 2023) with accession number GVM000082, including 28 wild emmer wheat and 13 durum wheat. CDS sequences of expansin gene family in wild emmer wheat and durum wheat were selected to map CDS sequences of high confidence genes set of bread wheat reference genome. Then, we extracted SNP loci information located in the corresponding CDS interval of the genes mapped in bread wheat from genotype dataset [61]. R package (geneHapR) was used to compute haplotype and haplotype frequencies.

### 4.7. Expression Analysis

For exploring the expression of family genes, RNA-seq data (SRR7226483, SRR7226484, SRR7226491, SRR2084160, SRR9025703, SRR7226462, and ERP022006) from EMBL-EBI database (https://www.ebi.ac.uk/; accessed on 9 January 2023) were collected and downloaded, including root, leaf, flower, glume, lemma, and grain in different plants of WEW and DW (Table S7) [33,34,49]. These data were analyzed by fastp v0.23.0 and hisat 2.2.1 software [61]. Obtaining BAM files were used to calculate the FPKM (Fragments Per Kilobase per million) values of genes using Rsubread package [62]. The heatmap were drawn using the R 4.2.1 according to the log2-transformed (FPKM + 1) values.

### 4.8. Validation of the Expression through qRT-PCR Analysis

To further observe the changes in gene expression in response to salt stress, we selected experimental materials with wild emmer (97 Yahudiya) and durum (Langdon) wheat. Firstly, seeds of these samples were sown for growth under hydroponic conditions, 22 ± 1 °C, 16 h light/8 h dark cycle in growth chamber. According to Han et al. who studied salt stress in wheat, 200 mM NaCl concentration was taken as salt stress treatment [31]. When the seedlings had been growing for three weeks, the roots from five plant samples of wild emmer (97 Yahudiya) and durum (Langdon) wheat were collected at 0 h, 0.5 h, 3 h, 8 h, and 27 h time points under salt treatment with three replicates each. RNA Easy Fast Plant Tissue Kit (Tiangen, Beijing, China) was used to extract total RNA from all samples and RT Master Mix Perfect Real-Time kit (Takara, Dalian, China) was used to synthesize cDNA according to the manufacturer’s instructions. qRT-PCR reaction was performed on a Quant-StudioTM 7 Flex System (Thermo Fisher Scientific, Waltham, MA, USA) using SYBR^®^ Green Premix Pro Taq HS qPCR Kit (Accurate Biology, Hunan, China) with the following thermal cycling conditions: 95 °C for 30 s followed by 40 cycles of 95 °C for 5 s, 60 °C for 34 s. All reactions were performed in three separate technological replicates. The expression levels of these 4 genes from expansin family were calculated using the 2^−ΔΔCT^ method with Taelf as the internal reference gene. The Ct values were used to represent relative expression. The primers used in this study are listed in the Supplementary Materials Table S8.

## 5. Conclusions

In summary, in this study, the expansin protein family was first identified at the genomic level in wild and durum wheat. First of all, we have identified 63 *TdEXPs* in wild wheat and 49 *TtEXPs* in durum wheat. The analysis of the physicochemical properties, exon–intron gene structure, and conserved motifs of the expansin genes showed that there were few differences in the physicochemical properties of the encoded proteins in the expansin family and the gene structure was relatively conserved. Next, the phylogenetic tree divides the expansin genes into the subfamilies EXPA, EXPB, and EXLA. Duplication and collinearity analysis showed that the expansin proteins of WEW and DW have higher ortholog, and the fragment duplication in the expansin family contributed to the amplification of the genes. Ka/Ks were in the range of 0.4173–0.9494 and were exposed to purifying selection during the evolution from WEW to DW. The codon usage analysis showed a preference for expansin proteins in WEW and DW, with a tendency to use synonymous codons ending in G or C, with C being used more frequently than G. Haplotype analysis identified five genes, and some haplotypes gradually became dominant during evolution for survival and improvement. We also studied that expansin genes show different expression patterns in different tissues and respond to salt stress. This study provides a vital clue for the evolution of this family in tetraploid wheat via the identification and evolutionary analysis of the expansins at the genome-wide level of WEW and DW, as well as a basis for the biological functions of the expansins and crop breeding in the future.

## Data Availability

Not applicable.

## Supplementary Materials

The following supporting information can be downloaded at: https://www.mdpi.com/article/10.3390/ijms241814120/s1.

## Abbreviations

WEW	Wild Emmer Wheat (*Triticum dicoccoides*)
DW	Durum Wheat (*Triticum turgidum* subsp. *durum*)
TdEXP	Expansin Gene Family in Wild Emmer Wheat (*Triticum dicoccoides*)
TtEXP	Expansin Gene Family in Durum Wheat (*Triticum turgidum* subsp. *durum*)
A3s	Adenine content at synonymous third codon position
C3s	Cytosine content at synonymous third codon position
G3s	Guanine content at synonymous third codon position
T3s	Thymine content at synonymous third codon position
GC3s	GC content at synonymous third codon position
