# Supplementary material for: Evolution and Expression of the Expansin Genes in Emmer Wheat
Source: Int J Mol Sci. 2023 Sep 15;24(18):14120. doi: 10.3390/ijms241814120 (PMC10531347; doi:10.3390/ijms241814120)
Supplement: Supplementary file 1 [file ijms-24-14120-s001.zip › Table S1 The sequence characterization of Expansin gene family in wild emmer wheat (Triticum dicoccoides).pdf]

Table S1. The sequence characterization of Expansin gene family in wild emmer wheat (*Triticum dicoccoides*).

Gene name	Gene ID	Protein ID	Chromosome Location (WEW_v2.1)	Protein length	PI	MW (Da)	GRAVY
TdEXP1	LOC119274440	XP_037411046.1	1A:377360942..377362450(+)	282	9.44	29427.46	0.004
TdEXP2	LOC119292586	XP_037427296.1	1A:382758584..382759811(-)	265	9.01	28503.79	-0.178
TdEXP3	LOC119275203	XP_037411905.1	1A:387137179..387142489(-)	397	6.76	42484.7	-0.353
TdEXP4	LOC119275230	XP_037411936.1	1A:387558165..387559684(-)	265	4.98	28714.09	-0.264
TdEXP5	LOC119275239	XP_037411942.1	1A:387920876..387923024(+)	278	6.93	30691.69	-0.321
TdEXP6	LOC119306056	XP_037438293.1	1B:419026522..419027911(-)	265	9.01	28581.86	-0.186
TdEXP7	LOC119338963	XP_037467060.1	1B:424876175..424877723(-)	270	6.50	29203.06	-0.147
TdEXP8	LOC119338984	XP_037467079.1	1B:425252028..425253707(-)	264	4.98	28627.01	-0.262
TdEXP9	LOC119339020	XP_037467106.1	1B:425381975..425384193(+)	276	6.94	30563.54	-0.324
TdEXP10	LOC119355777	XP_037478545.1	2A:618451801..618455926(-)	281	9.09	29355.52	-0.012
TdEXP11	LOC119351832	XP_037474522.1	2A:673847553..673848964(-)	281	9.17	29498.31	-0.125
TdEXP12	LOC119356467	XP_037479320.1	2A:709398854..709399964(+)	265	8.97	28852.14	-0.175
TdEXP13	LOC119364918	XP_037486386.1	2B:570094564..570098839(-)	281	8.89	29503.60	-0.043
TdEXP14	LOC119368162	XP_037489385.1	2B:634779652..634781078(-)	281	9.16	29494.34	-0.104
TdEXP15	LOC119368348	XP_037489533.1	2B:691305474..691307340(+)	265	9.14	28792.13	-0.174
TdEXP16	LOC119270920	XP_037408827.1	3A:10197605..10199556(+)	274	6.85	29857.93	-0.277
TdEXP17	LOC119270403	XP_037408303.1	3A:713997780..713999196(+)	319	4.95	33600.75	-0.246
TdEXP18	LOC119270404	XP_037408304.1	3A:714014191..714015475(+)	321	5.49	33352.39	-0.247
TdEXP19	LOC119270415	XP_037408316.1	3A:715476215..715477647(-)	321	5.50	33383.41	-0.256
TdEXP20	LOC119273456	XP_037410502.1	3A:719078911..719080405(+)	289	9.40	30150.62	-0.033
TdEXP21	LOC119280449	XP_037417188.1	3B:6812473..6814010(+)	291	6.13	31478.82	-0.268
TdEXP22	LOC119278538	XP_037415770.1	3B:796958403..796960009(+)	311	5.27	32476.47	-0.190
TdEXP23	LOC11928	XP_037418483.1	3B:799433867..7994	304	5.16	31780.55	-0.280

	2323		35288(-)				
TdEXP24	LOC119282360	XP_037418509.1	3B:805348619..805350167(+)	289	9.48	30277.98	0.023
TdEXP25	LOC119291882	XP_037426595.1	4B:629117226..629119275(+)	275	9.39	29527.00	-0.013
TdEXP26	LOC119294080	XP_037428242.1	4B:629404404..629405664(+)	271	5.75	28685.54	-0.023
TdEXP27	LOC119291886	XP_037426597.1	4B:629527603..629529281(+)	268	8.31	28615.61	-0.045
TdEXP28	LOC119294241	XP_037428365.1	4B:662242965..662244022(+)	270	8.02	29067.07	-0.417
TdEXP29	LOC119294247	XP_037428368.1	4B:663120257..663121346(+)	270	8.02	29022.96	-0.425
TdEXP30	LOC119294248	XP_037428369.1	4B:663136523..663137600(-)	270	8.31	29108.07	-0.441
TdEXP31	LOC119292244	XP_037426975.1	4B:671997469..671999765(-)	295	9.18	31855.24	-0.288
TdEXP32	LOC119292247	XP_037426979.1	4B:672288752..672290860(+)	343	5.80	35778.94	-0.318
TdEXP33	LOC119299575	XP_037432664.1	5A:88529123..88531130(-)	342	7.01	37555.74	-0.137
TdEXP34	LOC119303019	XP_037435994.1	5A:579907297..579910626(-)	308	5.27	32320.66	0.125
TdEXP35	LOC119303996	XP_037437060.1	5A:671107785..671109601(+)	275	9.50	29642	-0.054
TdEXP36	LOC119298377	XP_037431705.1	5A:671180708..671181997(+)	272	5.61	28785.8	0.082
TdEXP37	LOC119300405	XP_037433272.1	5A:671265341..671274039(+)	276	6.30	29215.21	-0.008
TdEXP38	LOC119303998	XP_037437061.1	5A:671333822..671335466(+)	273	8.78	28875.03	0.025
TdEXP39	LOC119298490	XP_037431770.1	5A:693373758..693374821(+)	270	8.02	28996.92	-0.414
TdEXP40	LOC119298496	XP_037431775.1	5A:693930635..693931772(+)	269	8.02	28937.89	-0.404
TdEXP41	LOC119304287	XP_037437363.1	5A:698902516..698904395(-)	341	5.91	35989.30	-0.328
TdEXP42	LOC119304289	XP_037437364.1	5A:699989006..699991711(+)	284	9.14	30771.90	-0.317
TdEXP43	LOC119307652	XP_037439618.1	5B:108110056..108112086(-)	341	7.50	37470.66	-0.094
TdEXP44	LOC119311692	XP_037443263.1	5B:570239552..570243117(-)	309	5.48	32456.80	0.090
TdEXP45	LOC119318828	XP_037449303.1	6A:16730809..16732063(-)	273	9.23	29766.71	-0.247
TdEXP46	LOC119318829	XP_037449304.1	6A:16752024..16753278(-)	273	9.23	29766.71	-0.247
TdEXP47	LOC119319043	XP_037449457.1	6A:16775209..16776571(-)	273	8.90	29815.70	-0.270
TdEXP48	LOC119318860	XP_037449328.1	6A:16801864..16803433(+)	273	9.31	29964.04	-0.254

TdEXP49	LOC119317359	XP_037447695.1	6A:474351759..474353348(-)	287	8.74	29956.11	-0.114
TdEXP50	LOC119317360	XP_037447696.1	6A:474589513..474591125(+)	289	9.45	30334.83	-0.118
TdEXP51	LOC119319382	XP_037449766.1	6A:474617338..474618907(-)	265	5.89	27780.19	-0.112
TdEXP52	LOC119324841	XP_037454504.1	6B:30135712..30136835(-)	273	9.28	29944.99	-0.286
TdEXP53	LOC119321215	XP_037450897.1	6B:30221094..30222483(-)	272	9.53	29901.11	-0.216
TdEXP54	LOC119324555	XP_037454236.1	6B:30231019..30232258(+)	273	9.33	29978.04	-0.226
TdEXP55	LOC119326789	XP_037456292.1	6B:437190737..437192134(-)	296	4.77	31467.30	-0.154
TdEXP56	LOC119326665	XP_037456184.1	6B:474729172..474733304(+)	268	8.99	28449.48	0.021
TdEXP57	LOC119321419	XP_037451006.1	6B:494803971..494805683(-)	287	8.85	30016.26	-0.083
TdEXP58	LOC119325403	XP_037455053.1	6B:495119935..495121548(+)	289	9.24	30294.67	-0.131
TdEXP59	LOC119343097	XP_037470171.1	Un(NW_021180253.1):1027..2330(+)	249	5.78	26831.52	-0.248
TdEXP60	LOC119344609	XP_037470891.1	Un(NW_021223648.1):94..1531(-)	253	8.31	26463.01	-0.020
TdEXP61	LOC119345468	XP_037471429.1	Un(NW_021249233.1):4..1307(-)	249	5.78	26831.52	-0.248
TdEXP62	LOC119347256	XP_037472009.1	Un(NW_021286646.1):1412..2882(+)	274	7.17	29917.02	-0.261
TdEXP63	LOC119347909	XP_037472293.1	Un(NW_021300509.1):1023..2326(+)	249	5.78	26831.52	-0.248
