# Supplementary material for: Evolution and Expression of the Expansin Genes in Emmer Wheat
Source: Int J Mol Sci. 2023 Sep 15;24(18):14120. doi: 10.3390/ijms241814120 (PMC10531347; doi:10.3390/ijms241814120)
Supplement: Supplementary file 1 [file ijms-24-14120-s001.zip › Table S2 The sequence characterization of Expansin gene family in durum wheat (Triticum turgidum).pdf]

Table S2. The sequence characterization of Expansin gene family in durum wheat (*Triticum turgidum*).

Gene name	Gene ID	Protein ID	Chromosome Location (Svevo.v1)	Protien length	PI	MW (Da)	GRAVY
TtEXP1	TRITD_1Av1G 134890	VAH06050.1	1A:363489604.. 363490576(-)	265	9.01	28503.79	-0.178
TtEXP2	TRITD_1Av1G 136420	VAH06168.1	1A:368023374.. 368028558(-)	397	7.09	42581.86	-0.351
TtEXP3	TRITD_1Av1G 136610	VAH06173.1	1A:368384529.. 368387394(-)	350	9.25	38890.61	-0.291
TtEXP4	TRITD_1Av1G 136660	VAH06176.1	1A:368490329.. 368491623(-)	248	4.84	26675.75	-0.240
TtEXP5	TRITD_1Bv1G 127340	VAH17760.1	1B:386796008.. 386796910(+)	275	9.50	28582.46	-0.020
TtEXP6	TRITD_1Bv1G 128930	VAH17884.1	1B:392974400.. 392975510(-)	265	9.01	28571.82	-0.183
TtEXP7	TRITD_1Bv1G 130670	VAH17988.1	1B:398680857.. 398682091(-)	281	8.17	30265.84	-0.096
TtEXP8	TRITD_1Bv1G 130750	VAH17995.1	1B:399057398.. 399058719(-)	264	4.98	28627.01	-0.262
TtEXP9	TRITD_2Av1G 019110	VAH25753.1	2A:36356017..3 6359578(-)	297	8.91	31165.37	-0.049
TtEXP10	TRITD_2Av1G 222160	VAH34300.1	2A:608884437.. 608888006(-)	281	8.99	29427.57	-0.023
TtEXP11	TRITD_2Av1G 243400	VAH35437.1	2A:665412410.. 665415862(-)	312	5.38	33053.59	-0.598
TtEXP12	TRITD_2Av1G 243410	VAH35439.1	2A:665419259.. 665434596(-)	281	9.17	29498.31	-0.125
TtEXP13	TRITD_2Av1G 259030	VAH36582.1	2A:700508959.. 700509870(+)	265	8.97	28852.14	-0.175
TtEXP14	TRITD_2Bv1G 184290	VAH49647.1	2B:544321683.. 544325275(-)	281	8.89	29513.63	-0.046
TtEXP15	TRITD_2Bv1G 204120	VAH50708.1	2B:609561374.. 609614447(-)	281	9.16	29494.34	-0.104
TtEXP16	TRITD_2Bv1G 221670	VAH51892.1	2B:667136912.. 667138548(+)	265	9.14	28792.13	-0.174
TtEXP17	TRITD_3Av1G 002370	VAH55612.1	3A:3904837..39 06132(-)	291	6.70	31661.19	-0.255
TtEXP18	TRITD_3Av1G 002390	VAH55613.1	3A:3947287..39 48542(-)	274	6.85	29783.79	-0.278
TtEXP19	TRITD_3Av1G 264150	VAH68957.1	3A:702298729.. 702316286(+)	319	4.85	33648.74	-0.260
TtEXP20	TRITD_3Av1G 264170	VAH68961.1	3A:702364587.. 702365639(+)	321	5.49	33384.44	-0.247
TtEXP21	TRITD_3Av1G 264880	VAH69016.1	3A:703737956.. 703739005(-)	302	5.32	31541.27	-0.263
TtEXP22	TRITD_3Av1G 266830	VAH69131.1	3A:707177302.. 707178487(+)	289	9.40	30150.62	-0.033
TtEXP23	TRITD_3Bv1G 003000	VAH70773.1	3B:5673418..56 74650(+)	274	7.17	29828.94	-0.270

TtEXP24	TRITD_3Bv1G003010	VAH70775.1	3B:5682013..5683358(+)	291	6.44	31774.17	-0.318
TtEXP25	TRITD_3Bv1G258860	VAH85002.1	3B:778479457..778480486(-)	311	5.27	32476.47	-0.190
TtEXP26	TRITD_3Bv1G261060	VAH85168.1	3B:785071863..785073054(+)	289	9.44	30230.76	-0.017
TtEXP27	TRITD_4Bv1G185720	VAI10271.1	4B:621399958..621401392(+)	275	9.39	29527	-0.013
TtEXP28	TRITD_4Bv1G185860	VAI10278.1	4B:621696982..621861807(+)	271	8.28	28897.93	-0.055
TtEXP29	TRITD_4Bv1G199480	VAI11125.1	4B:656286007..656286819(+)	270	8.02	29022.96	-0.425
TtEXP30	TRITD_4Bv1G202590	VAI11391.1	4B:662952440..662954224(+)	292	9.18	31684.08	-0.287
TtEXP31	TRITD_4Bv1G203220	VAI11452.1	4B:664300001..664301720(-)	341	5.80	35604.78	-0.315
TtEXP32	TRITD_5Av1G037950	VAI13686.1	5A:87266261..87267784(-)	342	7.01	37555.74	-0.137
TtEXP33	TRITD_5Av1G240040	VAI24733.1	5A:624380019..624381563(+)	294	10.69	31526.27	-0.217
TtEXP34	TRITD_5Av1G252100	VAI25642.1	5A:653104455..653105678(-)	161	4.80	17762.89	-0.266
TtEXP35	TRITD_5Av1G252510	VAI25650.1	5A:654181679..654183784(+)	284	9.04	30772.89	-0.317
TtEXP36	TRITD_5Bv1G034910	VAI28019.1	5B:97070838..97072445(-)	341	7.50	37470.66	-0.094
TtEXP37	TRITD_6Av1G006210	VAI41821.1	6A:14053180..14054678(-)	301	-	-	-0.341
TtEXP38	TRITD_6Av1G006600	VAI41862.1	6A:14873134..14874437(-)	273	9.23	29921.91	-0.262
TtEXP39	TRITD_6Av1G006660	VAI41864.1	6A:14993380..14994413(+)	273	9.46	29837.82	-0.263
TtEXP40	TRITD_6Av1G136030	VAI46788.1	6A:396990415..396991422(+)	297	4.71	31640.45	-0.157
TtEXP41	TRITD_6Av1G158520	VAI47825.1	6A:458944951..458946119(-)	287	8.74	29956.11	-0.114
TtEXP42	TRITD_6Av1G158640	VAI47826.1	6A:459184425..459185602(+)	289	9.45	30322.78	-0.136
TtEXP43	TRITD_6Bv1G009330	VAI53174.1	6B:24902745..24903925(-)	273	9.65	30047.12	-0.281
TtEXP44	TRITD_6Bv1G009410	VAI53177.1	6B:25037718..25038790(+)	273	9.38	29944.05	-0.214
TtEXP45	TRITD_6Bv1G009420	VAI53180.1	6B:25107433..25158362(+)	273	9.28	29913.92	-0.267
TtEXP46	TRITD_6Bv1G128400	VAI58270.1	6B:415352125..415353129(-)	296	4.77	31467.30	-0.154
TtEXP47	TRITD_6Bv1G138270	VAI58919.1	6B:453206534..453210161(+)	220	8.58	22945.21	0.165
TtEXP48	TRITD_6Bv1G144950	VAI59266.1	6B:473053959..473055168(-)	287	8.85	30016.26	-0.083
TtEXP49	TRITD_6Bv1G	VAI59267.1	6B:473361174..	289	9.24	30294.67	-0.131

145040	473362352(+)

”-” showed no results.
